# Combining advanced submucosal technologies for a fibrotic tattooed colonic recurrence

**DOI:** 10.1055/a-2803-5024

**Published:** 2026-03-16

**Authors:** Roberto De Sire, Antonio Capogreco, Francesco Minini, Davide Massimi, Cesare Hassan, Roberta Maselli, Alessandro Repici

**Affiliations:** 1 9268 Digestive Endoscopy Unit, IRCCS Humanitas Research Hospital, Rozzano, Milan, Italy; 2 9307 Gastroenterology, IBD Unit, Department of Clinical Medicine and Surgery, Università degli Studi di Napoli Federico II, Napoli, Italy; 3 437807 Department of Biomedical Sciences, Humanitas University, Pieve Emanuele, Milan, Italy

Lesions with prior manipulation, such as recurrences or those involving tattoo ink, are technically demanding due to submucosal fibrosis, obscured planes, and impaired lifting 1 . These challenges increase the risk of incomplete resection and adverse events. Endoscopic submucosal dissection (ESD) is often the optimal treatment strategy to achieve curative resection and avoid surgery.

We report the case of an 82-year-old woman with a 30 mm recurrent lesion at the hepatic flexure, extending hemi-circumferentially, following two previous hot endoscopic mucosal resections. Tattoo ink extended directly under the lesion, and prior histology had revealed tubular adenoma with high-grade dysplasia.

The procedure was performed using an underwater technique with continuous saline immersion, which enhanced visualization and provided natural traction 2 . A thin needle knife with high-pressure waterjet function was employed to facilitate dissection in the fibrotic and tattooed areas 3 . Despite these measures, limited scope maneuverability resulted in an intraprocedural perforation of the muscular wall, which was immediately closed with one through-the-scope clip. To optimize exposure, we applied a double rubber-band traction, which provided stable countertraction and facilitated continued dissection 4 .

Additionally, Amber-red color (ACI) imaging on the novel Fujifilm ELUXEO 8,000 processor was used to enhance contrast between vascular and muscular structures, even in the presence of dense tattoo ink, allowing for a clearer delineation of the subtle submucosal plane. 5 The lesion was successfully removed en bloc. The 35 × 25 mm specimen histology revealed a tubular adenoma with high-grade dysplasia with R0 resection. The mucosal defect was closed with a combination of a Mantis Clip (Boston Scientific) and additional through-the-scope clips to prevent delayed complications ( Video 1 ).

The combined use of underwater dissection, thin-needle high-pressure waterjet knife, traction, and ACI for a safe and curative resection of a fibrotic tattooed colonic recurrence. ACI, Amber-red color.Video 1

This case illustrates that tattooed and fibrotic recurrences represent one of the most challenging scenarios in ESD. The combined use of underwater dissection, thin-needle high-pressure waterjet knife, traction, and ACI can enable safe and curative resections in these sub-optimal settings.

Endoscopy_UCTN_Code_TTT_1AQ_2AD_3AD
